# Lysine Acetylome of Breast Cancer-Derived Small Extracellular Vesicles Reveals Specific Acetylation Patterns for Metabolic Enzymes

**DOI:** 10.3390/biomedicines11041076

**Published:** 2023-04-02

**Authors:** Zoran Minic, Yingxi Li, Nico Hüttmann, Gurcharan K. Uppal, Rochelle D’Mello, Maxim V. Berezovski

**Affiliations:** 1 John L. Holmes Mass Spectrometry Facility, Faculty of Science, University of Ottawa, Ottawa, ON K1N 6N5, Canada; 2 Department of Chemistry and Biomolecular Sciences, University of Ottawa, Ottawa, ON K1N 6N5, Canada

**Keywords:** breast cancer, protein acetylation, small extracellular vesicles, aldolase (ALDOA), glyceraldehyde-3-phosphate dehydrogenase (GAPDH), phosphoglycerate kinase (PGK 1), enolase (ENO), pyruvate kinase M1/2 (PKM)

## Abstract

Cancer-derived small extracellular vesicles have been proposed as promising potential biomarkers for diagnosis and prognosis of breast cancer (BC). We performed a proteomic study of lysine acetylation of breast cancer-derived small extracellular vesicles (sEVs) to understand the potential role of the aberrant acetylated proteins in the biology of invasive ductal carcinoma and triple-negative BC. Three cell lines were used as models for this study: MCF10A (non-metastatic), MCF7 (estrogen and progesterone receptor-positive, metastatic) and MDA-MB-231 (triple-negative, highly metastatic). For a comprehensive protein acetylation analysis of the sEVs derived from each cell line, acetylated peptides were enriched using the anti-acetyl-lysine antibody, followed by LC-MS/MS analysis. In total, there were 118 lysine-acetylated peptides, of which 22, 58 and 82 have been identified in MCF10A, MCF7 and MDA-MB-231 cell lines, respectively. These acetylated peptides were mapped to 60 distinct proteins and mainly identified proteins involved in metabolic pathways. Among the acetylated proteins identified in cancer-derived sEVs from MCF7 and MDA-MB-231 cell lines are proteins associated with the glycolysis pathway, annexins and histones. Five acetylated enzymes from the glycolytic pathway, present only in cancer-derived sEVs, were validated. These include aldolase (ALDOA), glyceraldehyde-3-phosphate dehydrogenase (GAPDH), phosphoglycerate kinase (PGK1), enolase (ENO) and pyruvate kinase M1/2 (PKM). For three of these enzymes (ALDOA, PGK1 and ENO) the specific enzymatic activity was significantly higher in MDA-MB-231 when compared with MCF10A-derived sEVs. This study reveals that sEVs contain acetylated glycolytic metabolic enzymes that could be interesting potential candidates for early BC diagnostics.

## 1. Introduction

Extracellular vesicles (EVs), derived from human cancer cells, play a role in driving various cellular processes related to cancer biology, such as the modulation of tumor microenvironment, angiogenesis, sustained growth and tissue invasion and metastasis [1,2,3,4,5,6,7,8]. EVs carry a variety of molecules (nucleic acids, proteins and metabolites) that can be released from the donor to nearby or distant cells [8,9,10,11]. Consequently, EV exchange between cells appears as a crucial modulator of cell–cell communication and could be an important player in health and disease states. Membrane-bound EVs are classified, based on their distinct biogenesis pathways, into three main subtypes: exosomes, microvesicles and apoptotic bodies. Additionally, EVs can also be classified based on their substantially different sizes. Small extracellular vesicles (sEVs) are in the range of 30 to 150 nm [12,13], while medium extracellular vesicles (mEVs) are in the range of 100 nm to 1000 nm in diameter [14,15]. In our study, we isolated vesicles in the range of 50–300 nm in diameter, referred to as sEVs.

EVs are of great interest for molecular biomarker discovery that enable monitoring cancer progression as well as early diagnosis and accurate prognosis of BC. In our previous investigations, using proteomics approaches, numerous proteins have already been identified from EVs as potential biomarkers for early diagnosis or as therapeutic targets [7,8]. Moreover, using a phosphoproteomics approach, we previously assessed the enzymatic activity of three enzymes associated with cancer and only present in sEVs derived from the cancerous cell lines MCF7 and MDA-MB-231: ATP citrate lyase (ACLY), sirtuin-1 (SIRT1) and sirtuin-6 (SIRT6) [16]. The specific activity of these enzymes was significantly higher in MDA-MB-231 sEV fractions when compared with similar MCF10A fractions. Our previous work demonstrated the presence of functional metabolic enzymes in sEVs specific to BC cells that may be further explored for early BC diagnostics. Furthermore, this investigation also indicates that post-translational modifications (PTMs) of EV proteins can be a valuable resource for biomarker candidates. Enzymes related to the NAD+ deacetylase group, such as sirtuin-1 (SIRT1) and sirtuin-6 (SIRT6), can have several different functions [17,18,19,20,21,22,23,24,25,26]. SIRT1 has been shown to participate in apoptosis, autophagy, senescence, proliferation, aging, tumorigenesis, genome regulation, stability and maintenance [17,18,19,20,21,22,23]. SIRT6 also affect several processes and is involved in DNA repair, glycolysis, gluconeogenesis, tumorigenesis, neurodegeneration and cardiac hypertrophic responses [24,25,26]. The presence of these enzymes in sEVs questions the role of protein acetylation in the regulation of BC progression and tumor growth.

Protein acetylation is a reversible PTM in which the acetyl group from acetyl coenzyme A (Ac-CoA) is transferred to either the α-amino group of the protein’s N-terminus or to the ε-amino group of lysine residues [27]. The lysine acetylation of proteins is catalyzed by lysine acetyltransferases (KATs), and this PTM is reversible and regulated by enzymes, namely deacetylases (KDACs), that can remove the acetyl group [28,29]. Acetylation results in the neutralization of the positive charge on the lysine residue and consequently alters protein function [28,29]. Interestingly, some studies have reported that lysine acetylation in mitochondria, where a high concentration of acetyl-CoA and an elevated pH are present, can also occur in a non-enzymatic manner [30,31].

By using proteomic approaches, 181 and 244 acetylation sites have been identified in human BC MDA-MB-231 cells from enrichment with the monoclonal antibody cocktail and the polyclonal antibody, respectively [32]. The overall acetylome revealed that the acetylation levels of the majority of proteins in BC tissue were significantly higher than those in normal tissue [33]. This study revealed that highly acetylated proteins were significantly enriched in histone H2A.X (H2A.X) complexes and nucleophosmin (NPM1). Atypically acetylated proteins have been shown to promote breast cancer metastasis and the proliferation of breast cancer [34,35,36,37,38,39,40]. Moreover, acetylation can inhibit the sensitivity of tumor cells to anti-tumor therapy oncogenesis and progression of BC [41,42]. The regulation of acetylated proteins such as CREB binding protein (CBP), ALDH1A1, α-tubulin, cortactin and Forkhead Box O3 (FOXO3) show tumor-suppressing effects in BC [43,44]. It has been demonstrated that inhibitors targeting protein acetylation can be used as potential drug candidates [34].

Reversible lysine acetylation acts as an indispensable regulator in multiple cellular pathways, oncogenesis and progression of BC. To date, the potential role of this PTM in extracellular vesicles related to cancer and more specifically to BC is still unknown. The fact that protein acetylation can affect protein function will elicit interest in exploring and evaluating this PTM for early diagnosis of BC. This study explored the lysine acetylome of sEVs derived from metastatic BC cell lines, MDA-MB-231 and MCF7, and the non-cancerous breast tissue cell line MCF10A. Differences in EV acetylomes were observed between the non-cancerous and cancerous breast cell lines. We selected and tested the enzymatic activity of five acetylated enzymes: aldolase (ALDOA), glyceraldehyde-3-phosphate dehydrogenase (GAPDH), phosphoglycerate kinase (PGK 1), enolase (ENO) and pyruvate kinase M1/2 (PKM). ALDOA, PGK 1 and ENO had significantly higher specific enzymatic activities in MDA-MB-231 compared to MCF10A-derived sEVs.

## 2. Materials and Methods

### 2.1. Cell Culturing and sEV Isolation

Culturing of epithelial breast cancer cell line MDA-MB-231 (ATCC HTB-26), MCF7 (ATCC HTB-22) and MCF10A non-tumorigenic epithelial breast cancer cell line (ATCC CRL-10317) was performed as described previously [7,8,16]. sEVs were isolated by using differential ultracentrifugation as described in our previous investigation [7,16].

### 2.2. Quantification of sEVs by Nanoparticle Tracking Analysis (NTA)

The concentration and size distribution of sEVs were determined using The ZetaView nanoparticle tracking microscope PMX-110 (Particle Metrix, Meerbusch, Germany) at 85 and 40 camera shutter speeds [16].

### 2.3. Characterization of EVs Protein Markers

Marker-based assessment of EVs isolated from MCF10A, MCF7 and MDA-MB-231 cell lines was performed using the commercially available Exo-Check Exosome Antibody Array kit (System Biosciences, Palo Alto, CA, USA) according to the manufacturer’s protocol.

### 2.4. Sample Preparation for Acetylomics

Isolated sEVs were added in lysis buffer with a volume ratio of 4:1 (fraction/buffer) consisting of a final concentration of 20 mM HEPES, pH 8.0, 0.1% NP-40, 1 mM DTT, 1.6 M urea, 1/1000 (*v*:*v*) protease inhibitor cocktail (Cat No. 78430, Thermo Fischer Scientific, Mississauga, ON, Canada), 3 µM Trichostatin A (TSA) and 10 mM Nicotinamide (NAM) and were gently vortexed for 2 min. The suspension was centrifuged for 3 min at 10,000x *g* and the supernatant was then collected. The protein concentration was determined using a Bradford protein assay kit (Thermo Scientific, Cat No. 23236, Waltham, MA, USA).

### 2.5. Protein Reduction, Alkylation and Enzymatic Digestion

Protein samples obtained from the isolated sEVs were reduced, alkylated and digested using FASEB method as described previously [45]. Proteolytic digestion was performed by addition of sequencing grade modified trypsin (Promega, #V5111, Madison, WI, USA), 1:300 enzyme to protein ratio, and incubated under shaking at 500 rpm at ambient temperature overnight. The digestion was stopped by addition of formic acid (1% final concentration) and centrifuged at 15,000× *g* for 3 min. The supernatant containing about 100 µg of digested protein was desalted on disposable TopTip C-18 columns (Glygen, #TT2C18.96, Ellicott City, MD, USA) and dried by vacuum centrifugation.

### 2.6. Enrichment of Acetylated Peptides

The peptides were dissolved in 100 µL of the immunoprecipitation buffer solution containing 50 mM HEPES (pH 8.0), 100 mM NaCl, 1 mM EDTA, 0.1% NP-40 and incubated with 30 µL pre-washed antibody beads (catalog no. PTM-104 for Kac, PTM Biolabs, Inc., Hangzhou, China) at 4 °C overnight with gentle shaking. The bound lysine-acetylated peptides were then processed according to the protocol of PTM Biolabs. The eluted peptides were collected and vacuum-dried followed by resuspension in 20 µL of 0.1% FA and analyzed by LC-MS/MS.

### 2.7. Nano-LC-MS/MS

An Orbitrap Fusion mass spectrometer (Thermo Fisher Scientific, Mississauga, ON, Canada) equipped with an UltiMate 3000 nanoRSLC (Thermo Fisher Scientific, Mississauga, ON, Canada) was used for nanoLC-MS/MS analysis. Three microliters of enriched acetylated peptides were loaded onto the column for 65 min at a flow rate of 0.30 μL/min and separated on an in-house packed column (Polymicro Technology, Phoenix, AZ, USA), 15 cm × 70 μm ID, Luna C18(2), 3 μm, 100 Å (Phenomenex) employing a water/ACN/0.1% formic acid gradient. The following steps were employed: 0–10 min, 2–2% ACN; 10–40 min, 2–38% ACN; 40–45 min, 38–98% ACN; 45–50 min, 98–98% ACN; 50–55 min, 98–2% ACN; 55–65 min, 2–2% ACN. Data-dependent MS/MS acquisition was performed following a full MS survey scan. The Orbitrap parameters in ESI+ were set up as follows: ion spray voltage 2.1 kV, ion source temperature 250 °C, top speed mode over the *m/z* range (*m/z* 350–2000) at a resolution of 60,000. Precursor ions were filtered according to monoisotopic precursor selection and charge state (+2 to +7), and dynamic exclusion was enabled for 30 s. The automatic gain control settings were 5 × 10^5^ for full scan and 1 × 10^4^ for MS/MS scans. Fragmentation was performed with collision-induced dissociation (CID) in the linear ion trap. Precursors were isolated using a 2 *m/z* isolation window and fragmented with a normalized collision energy of 35%.

### 2.8. MS Spectra Processing

Proteome Discoverer 2.1 (Thermo Fisher Scientific, Mississauga, ON, Canada) was used for protein identification. The precursor mass tolerance was set at 10 ppm and 0.6 Da mass tolerance for fragment ions. Search engine SEQUEST-HT implemented in Proteome Discoverer was applied for all MS raw files. Search parameters were set to allow for dynamic modification of methionine oxidation, lysine (K) acetylation, N-terminus acetylation and static modification of cysteine carbamidomethylation. Peptides were searched against a human UniProt FASTA file containing 20,396 entries (21 April 2021) and a default contaminants database. The false discovery rate (FDR) was set to 0.01 for both the protein and peptide level.

### 2.9. Data Filtering and Acetylation Site Localization

The SEQUEST-HT output table containing information on PSMs was used for all analysis in R [46]. Each cell line was cultured and analyzed in triplicate. Acetylated peptides which were found in at least two out of the three replicates were used for further analysis. Acetylated peptides were then collapsed to acetylation sites on unique proteins. Enriched motifs for acetylation sites per cell line were identified with motif-x. The biological function of acetylated proteins in each cell line were annotated with Gene Ontology (GO) and KEGG terms. Identified acetylation sites were compared with known acetylation sites downloaded from PhosphoSitePlus (20 October 2022) [47].

### 2.10. Disease and Functional Annotation Analysis

Gene ontology and KEGG functional annotations were obtained through the clusterProfiler R package [48,49,50]. Absolute numbers of proteins belonging to presented biological themes were compared between cell lines.

### 2.11. Data Availability

All MS raw data were submitted to the PRIDE repository (Accession: PXD040413) at the European Bioinformatics Institute.

### 2.12. Aldolase Activity Assay

Cells and isolated extracellular vesicles were resuspended in ice-cold Aldolase Assay Buffer from the kits (ab196994, Abcam, Toronto, ON, Canada) and gently vortexed. After centrifugation at 10,000× *g* for 1 min, supernatants were collected and used for enzymatic activities. The assays were performed at 37 °C, and absorbances were measured at 450 nm in a kinetic mode according to the manufacturer’s instructions. The activity of enzymes was calculated from the assay time of 30 min.

### 2.13. Glyceraldehyde 3 Phosphate Dehydrogenase Activity Assay

The glyceraldehyde 3 phosphate dehydrogenase activity was measured using a Glyceraldehyde 3 phosphate dehydrogenase Activity kit (ab204732, Abcam, Toronto, ON, Canada) according to the manufacturer’s procedures. The assays were performed in 100 µL of the reaction mixture on a microplate reader at 37 °C, and absorbances were measured at 450 nm in a kinetic mode based on the manufacturer’s instructions. The activity of enzymes was calculated from the assay time at 10 (cells) and 50 min (EVs).

### 2.14. Phosphoglycerate Kinase Activity Assay

The Phosphoglycerate Kinase Activity was assessed by using a colorimetric assay (The Phosphoglycerate Kinase Activity Assay Kit, ab252890, Abcam, Toronto, ON, Canada) following the manufacturer’s procedures. The assays were performed on a microplate reader (Greiner Bio-One 655209; Fischer Scientific, Toronto, ON, Canada) at 37 °C in a kinetic mode at 340 nm as described in the manufacturer’s instructions. The activity of enzymes was calculated from the assay time of 20 min.

### 2.15. Enolase Activity Assay

The protein extracts from cells and EVs were treated with enolase assay buffer (Abcam Enolase assay kit, ab241024, Abcam, Toronto, ON, Canada), and supernatants were collected after centrifugation at 10,000× *g* for 1 min. The assay was performed as indicated in the manufacturer’s instructions at 37 °C in a kinetic mode using a fluorometric method (Ex/Em = 535/587). The activity of enzymes was calculated from the assay time between 10 and 30 min.

### 2.16. Pyruvate Kinase Activity Assay

Briefly, the reaction was started by incubating the protein extract with the reaction mixture following the manufacturer’s instructions (Pyruvate Kinase Assay Kit, ab83432, Abcam, Toronto, ON, Canada). Samples were incubated at 37 °C in a kinetic mode, and the fluorescence was measured between 0 and 10 min using a microplate reader (Ex/Em = 535/587).

### 2.17. Statistical Analysis

Enzymatic assays were conducted at least in quadruplicates. Replicates of each cell line within a sample origin group, cell-free extract or sEVs, were compared for statistical significance of their means by a Student’s *t*-test. All *p*-values were annotated and a *p*-value of <0.05 was considered significant.

## 3. Results

### 3.1. Isolation of sEV

**Figure 1:**
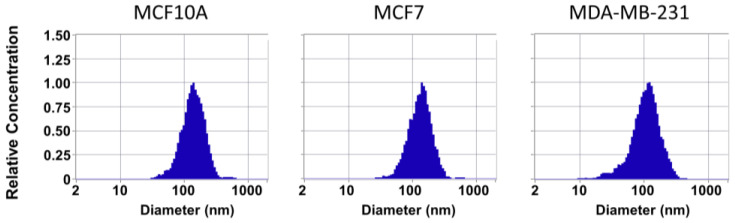
Nanoparticle tracking analysis (NTA) of extracellular vesicles. NTA plots show the size distribution profiles of isolated sEVs from MCF10A, MCF7 and MDA-MB-231 cell lines.

To explore the potential role of acetylated proteins for early BC diagnostics, we performed a systematic acetylome analysis of sEVs derived from MCF10A, MCF7 and MDA-MB-231 cell lines. Nanoparticle tracking analysis (NTA) was used to evaluate the size distribution of sEVs isolated by differential ultracentrifugation. The diameter of isolated sEVs ranged from 50 to 300 nm, with an average size of about 125 nm (Figure 1). Additionally, the ExoCheck kit validated the presence of several external (CD63, EpCAM, ANXA5, CD81 and ICAM) and internal (TSG101, ALIX and FLOT1) EV markers in isolates from all three cell lines (Figure S1).

### 3.2. Overall Acetylome Profiling

**Figure 2:**
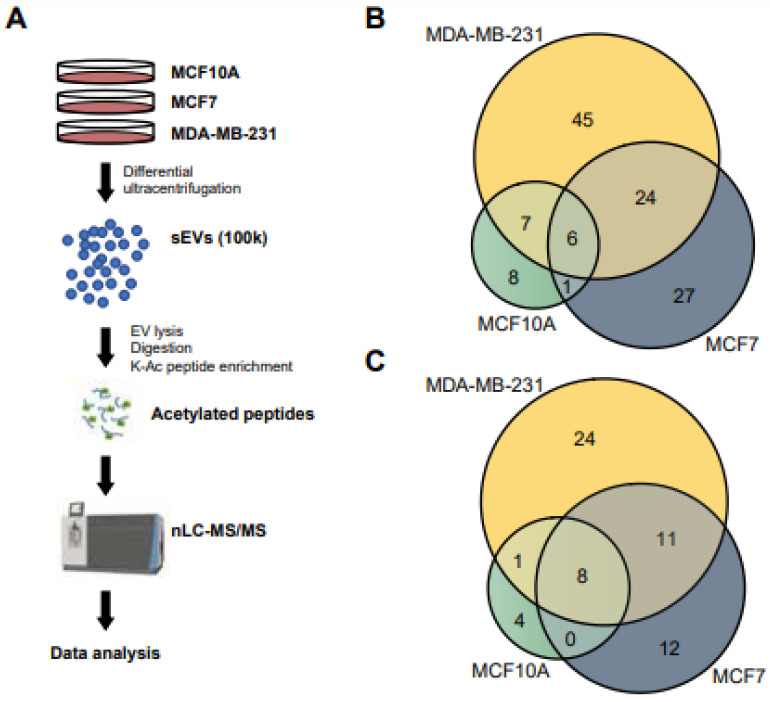
Overview of the identified acetylated peptides/proteins. (**A**) Workflow illustration of acetylome analysis of sEVs from MCF10A, MCF7 and MDA-MB-231 cells. (**B**) Total number of acetylated peptides identified from three independent replicates of each cell line (**C**) A Venn diagram showing the number of EV acetylated proteins identified from the three cell lines.

Samples of about 100 µg from MCF10A, MCF7 and MDA-MB-231 derived sEVs collected from three biological replicates were prepared and examined independently. Proteins were digested and the acetylated peptides were enriched using anti-acetyl-lysine (KAC) immunoaffinity chromatography to enhance the identification of low-abundance acetylated proteins. A schematic overview of our experimental strategy is presented in Figure 2A. After harvesting and isolating sEVs using ultra-centrifugation, acetylated peptides were enriched then analyzed by nano liquid chromatography-tandem mass-spectrometry (nLC-MS/MS) using CID fragmentation mode. The proteomic mass spectrometry data were subjected to rigorous bioinformatics assessment and protein identification related to lysine acetylation. Acetylated peptides were considered reliable if they were identified in at least two biological replicates. After removal of potential contaminants like keratins, the total number of identified acetylated peptides for each cell line is presented in Figure 2B and Table S1. In total, from 118 acetylated peptides 22, 58 and 82 have been identified in MCF10A, MCF7 and MDA-MB-231sEVs, respectively (Figure 2B). The Venn diagram of acetylated proteins revealed in total 60 identified proteins from three cell lines including 13, 41 and 44 acetylated proteins from MCF10A, MCF7 and MDA-MB-231 sEVs, respectively (Figure 2C and Table S2).

### 3.3. Acetylation Site Distributions and Motifs for the Identified Acetylation Sites

**Figure 3:**
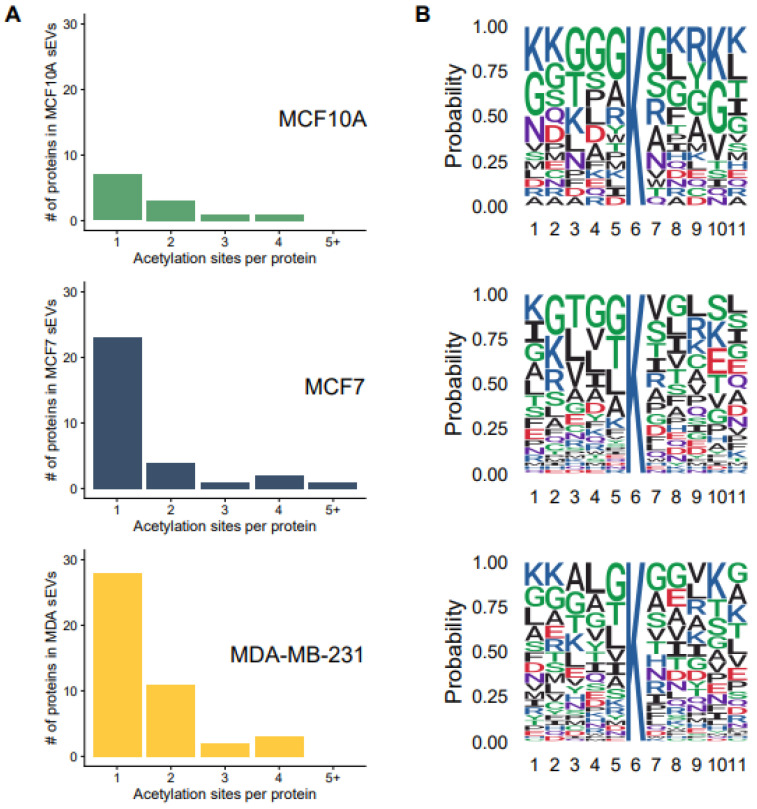
Overview of the acetylated sites and acetylation motifs. (**A**) Barplot depicting the distribution of numbers of acetylation sites observed per protein in all three EV-derived cell lines. (**B**) Conservation of acetylation motif for ±10 amino acids around the lysine acetylation sites. The central K refers to the acetylated lysine and the size of each letter corresponds to the frequency of the amino acid residue in that position.

The distribution of acetylated residues per protein in sEVs derived from three breast cell lines is presented in Figure 3A. Among the 97 identified sites, 25 are not previously reported (Table S3). A single acetylation site was localized on most of the identified acetylated proteins in all three sEV fractions. A notable number of the identified proteins were found to be lysine acetylated at two or more sites. MCF7 (eight acetylated proteins) and MDA-MB-231 (16 acetylated proteins) derived sEVs contained a higher number of multiply acetylated proteins than MCF10A (five acetylated proteins) derived particles (Figure 3A). Some proteins contained five or more acetylated sites such as fatty acid synthase (FASN) and H4 clustered histone 9 (H4C9).

Visualized sequence motifs from the acetylation for each cell lines derived sEV are presented in Figure 3B. Acetylation motives have not revealed substantially different motifs for lysine acetylation from all EV fractions. It appears that G at the −1 and −2 positions as well as K and G at the +4 position are overrepresented in MCF10A in comparison to MCF7 and MDA EVs. Some variations of amino acids were observed at the position from +1 to +5 in all three EV fractions. Therefore, these results may suggest different acetylation dynamics between the three cell lines.

### 3.4. Functional and Pathway Analysis of Identified Acetylated Proteins

**Figure 4:**
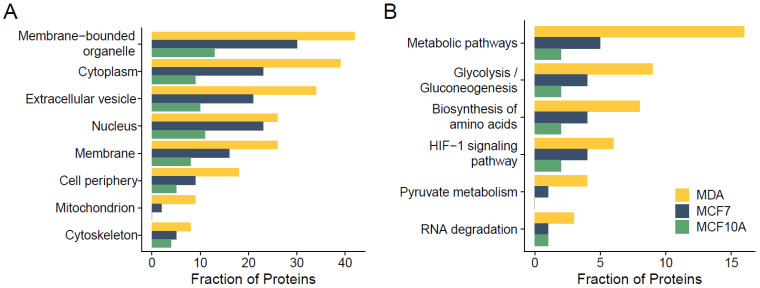
GO cellular localization (**A**) and KEGG pathways analysis (**B**) of acetylated proteins derived from MCF10A, MCF7 and MDA-MB-231 sEVs.

**Figure 5:**
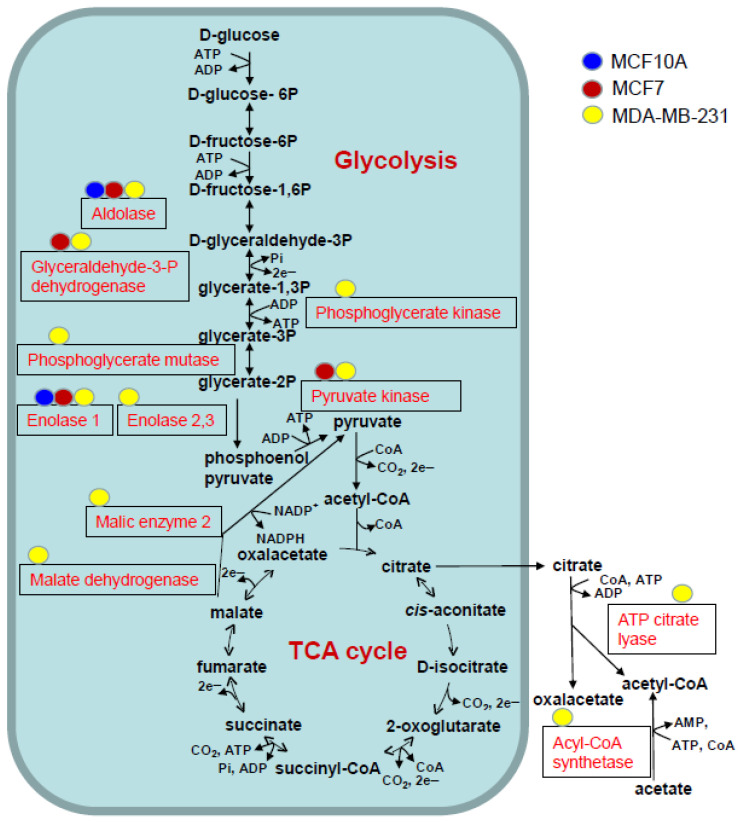
The enrichment of acetylated enzymes in the glycolysis pathway and TCA cycle in breast cancer cell lines. The acetylated enzymes from MCF10A, MCF7 and MDA-MB-231 are marked in blue, red and yellow respectively.

Identified acetylated proteins were annotated and classified using the Gene Ontology (GO) and Kyoto Encyclopedia of Genes and Genomes (KEGG) databases. Within the GO subcellular localization terms, the identified lysine-acetylated proteins were mainly localized to the membrane-bounded organelle, cytoplasm, extracellular vesicle, nucleus and membrane (Figure 4A). KEGG enrichment demonstrated that many proteins were associated with the terms of metabolic pathways, glycolysis/gluconeogenesis, biosynthesis of amino acids, HIF-1 signaling pathway and pyruvate metabolism (Figure 4B). For all these terms, a larger number of proteins was presented in MCF7 and MDA-MB-231 derived sEVs, in comparison to MCF10A derived sEVs. Remarkably, the majority of acetylated proteins were associated to the glycolysis pathway (Figure 5). 

A noteworthy number of lysine-acetylated proteins from MCF7 and MDA-MB-231 derived sEVs were observed for annexins (Figure S2). Numerous acetylated histones were identified among different cell line derived sEVs including H4 clustered histone 9, H3 clustered histone 1, H3.4 histone, H2B clustered histone 18, H2B clustered histone 20 and H1.5 linker histone (Table S2). Interestingly, KAT8 regulatory NSL complex subunit 1, which was identified in this study, is known to be involved in the acetylation of nucleosomal histone H4 in various lysed residues [51].

Our previous work focused on identifying metabolic enzymes in BC-derived EVs and MVs that could be explored as diagnostic BC biomarker [8,16]. We chose to focus on enzymes as biomarkers since they can be easily incorporated into quick, accurate and sensitive diagnostic assays that can assess specific enzymatic activity. sEV enzymes annotated by the KEGG database with the term “glycolysis/metabolic pathways” were selected for downstream experiments. We investigated five enzymes (Table 1, Figure S3) mainly acetylated in MCF7 and/or MDA-MB-231 sEVs: aldolase (ALDOA), glyceraldehyde-3-phosphate dehydrogenase (GAPDH), phosphoglycerate kinase (PGK1), enolase (ENO) and pyruvate kinase M1/2 (PKM). We did not perform further validation for phosphoglycerate mutase 1 (PGAM1) because of the unavailability of a kit capable of measuring its enzymatic activity.

### 3.5. Analysis of ALDOA, GAPDH, PGK 1, ENO and PKM in Cells and Their sEVs

**Figure 6:**
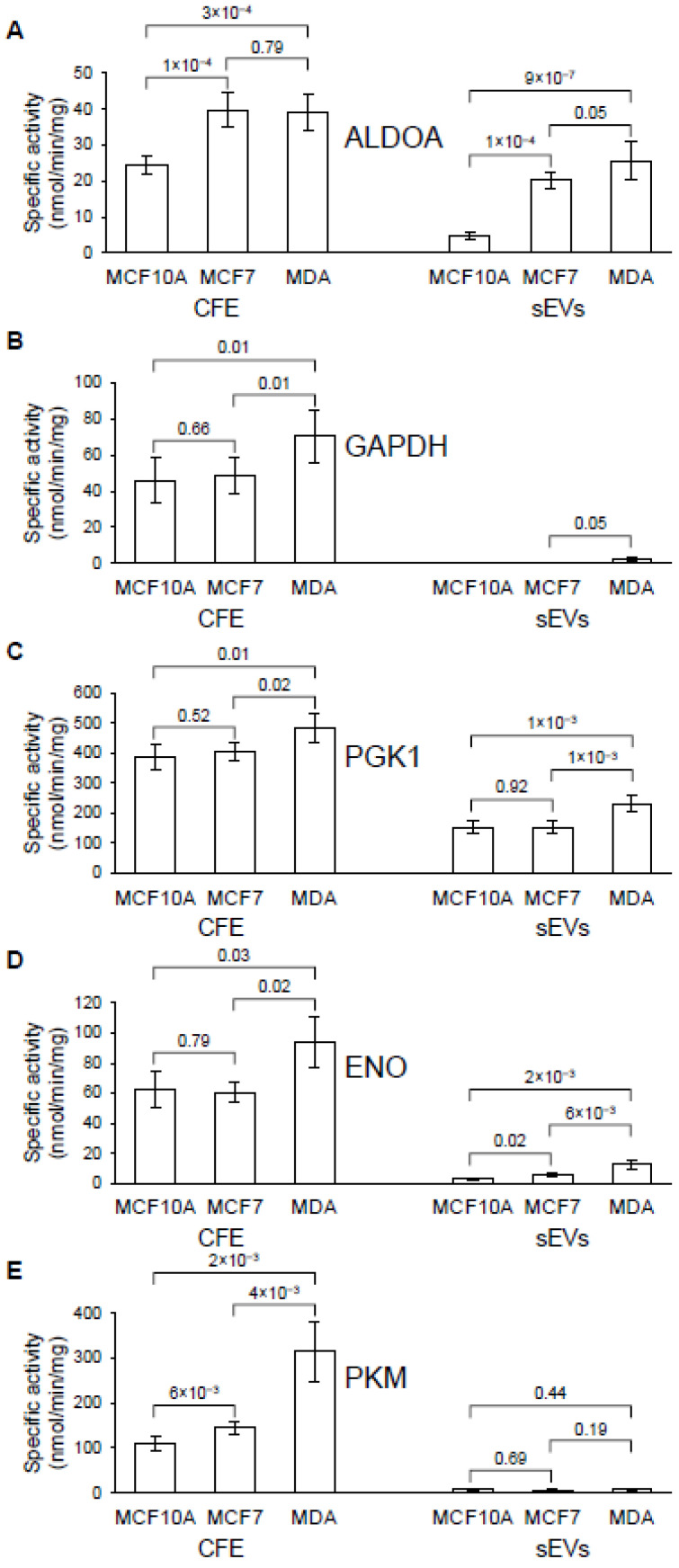
Specific enzymatic activity of (**A**) aldolase (ALDOA), (**B**) glyceraldehyde-3-phosphate dehydrogenase (GAPDH), (**C**) phosphoglycerate kinase (PGK1), (**D**) enolase (ENO) and (**E**) pyruvate kinase M1/2 (PKM) in cell-free extract (CFE) and their corresponding sEV fractions. The bar graph represents mean values, while error bars indicate the standard deviation (SD) of at least five replicates, and *p*-values obtained from a Student’s t-test are indicated on the top.

The enzymatic activity was detected in all extracted protein fractions from the three cell lines (Figure 6, Table S5). The specific activity levels of all tested enzymes are significantly higher in MDA-MB-231 when compared with MCF10A cell line. Similarly, the specific activities of ALDOA and ENO were significantly higher in MDA-MB-231 and MCF7 when compared with MCF10A cell derived sEVs. The specific activity of PGK1 was significantly higher in MDA-MB-231 in comparison to MCF10A and MCF7 sEV fractions. Very low or absence of activity was observed for GAPDH and PKM in all EV fractions. Although some activity of GAPDH was observed in MDA-MB-231 and MCF7 from sEV in comparison to MCF10A, the results cannot be conclusive because of very low specific activity. Therefore, ALDOA and ENO reveal a more significant difference in specific activity between sEV derived from MCF10A and MCF7 or MDA-MB-231.

## 4. Discussion

In recent years, proteomics has played a remarkable role in providing information about EVs and their function in breast cancer tumor progression and metastasis [2,3,4,5,6,7,8]. Post-translational modification of proteins intersects with cancer, playing a decisive role in regulating various cellular processes and being implicated in the major regulatory mechanisms of cell signaling networks. In our previous investigation, using proteomics analysis, we demonstrated that EVs contain functional, phosphorylated, metabolic enzymes that could be potential candidates in early BC diagnostic and therapeutic applications [16]. In this study, 25 novel acetylated sites were identified in sEVs from two breast cancer cell lines (MCF7 and MDA-MB-231) and the non-metastatic (MCF10A) cell line. These acetylated sites were mapped to 60 distinct proteins. The cancerous cell lines used in this work present a good model for studying invasive ductal carcinoma (MCF7) and triple-negative BC (MDA-MB-231)

Although enrichment analysis was performed using the same initial quantity of proteins, about 100 µg from all three sEVs, there was a significant difference in the number of identified acetylated proteins isolated from these three cell lines. Therefore, EVs from MCF10A produced a lower number of lysine acetylated proteins in comparison to MDA-MB-231 and MCF7. This finding suggests that lysine acetyltransferase enzymes are working more effectively in cancerous cell lines. Some of these enzymes have been overexpressed in various types of cancers such as colon, liver, glioma, bone, lung, breast and prostate cancers [52,53]. Accordingly, some studies have explored small-molecule inhibitors from the KAT protein family for cancer therapy [53]. In this study, numerous identified acetylated proteins in sEV-derived BC cell lines indicated that their modification could affect protein function and holds a biological role and prognostic value in breast cancer.

The majority of acetylated proteins were identified to participate in various metabolic processes such as glycolysis, biosynthesis of amino acids and pyruvate metabolism. It has been investigated that cancer cells are able to reprogram different metabolic processes to meet requirements for proliferation, invasion and survival in hostile environments [54,55]. Glucose is one of the primary fuel sources for malignant cells and plays an important role in tumorigenic metabolism and cancer progression [56]. Therefore, it is not surprising that several identified acetylated proteins are associated with the glycolysis pathway. During the process of oncogenesis, an essential piece of metabolic reprogramming of cancerous cells is enhancing aerobic glycolysis and glucose uptake [57,58]. Five enzymes involved in glycolysis present only or mainly in sEVs isolated from cancer-derived cell lines were validated: aldolase (ALDOA), glyceraldehyde-3-phosphate dehydrogenase (GAPDH), phosphoglycerate kinase (PGK1), enolase (ENO) and pyruvate kinase (PKM). However, only three of these enzymes, ALDOA, PGK1 and ENO, have a higher value for specific enzymatic activity in MDA-MB-231 derived sEVs when compared with MCF10A ones. Several investigations demonstrated increasing activity/expression of these enzymes in breast cancer cells or tissues and are proposed as promising breast tumor markers. The enzyme activities of ALDOA and ENO were found to significantly increase in cancerous breast tissue when compared to normal tissue [59]. The overexpression of phosphoglycerate kinase has been demonstrated in most cancer types, including BC [60]. This study validated that PGK1 can be used for BC prognosis. In the present work, we found that the activities of ALDOA, PGK1 and ENO were increased in BC derived sEVs isolated from the MDA-MB-231 cell line, in comparison to sEVs isolated from MCF10A cell lines. These findings suggest that sEV enzymes may be potential clinical indicators for the diagnosis of BC.

The presence of metabolite products from identified acetylated enzymes from the glycolysis pathway and malate dehydrogenase from the TCA cycle can serve as precursors for amino and fatty acid synthesis [61]. This finding suggests that pathways for amino and fatty acid synthesis were affected with lysine acetylation. This is not surprising because it is known that in addition to glucose, tumor cells metabolize amino and fatty acids at much higher rates than their nontumor equivalents [62].

In this study, we also identified four acetylated annexin proteins (annexin 1, 2, 5 and 6) present in cancerous cell line derived sEVs. Annexin family members can regulate various cellular functions including vesicle trafficking, vesicle fusion, plasma membrane repair, promotion of membrane segregation and actin cytoskeleton dynamic regulation [63,64,65,66]. Dysregulation of annexins has been associated with multiple cancers including BC, and these proteins have emerged as potential biomarkers and pharmacological targets for medical applications [67]. Quantitative mass spectrometry-based proteomic analyses revealed significantly higher ANXA1 protein expression in more invasive glioblastoma cells [68]. The abundance of ANXA1 was significantly higher in the high-grade glioblastoma EVs compared with low-grade glioblastoma EVs [69]. Therefore, this protein has been proposed as a potential EV biomarker for glioblastoma. In a model of human BC, ANXA1 can promote tumor-formation, and it has been suggested that some basal-like TNBCs may require high endogenous tumor cell Annexin A1 expression for continued growth [70]. In addition, ANXA1 has been suggested to be a useful prognostic marker in HER-2+ BC patients [71]. The protein Annexin A2 has been explored as a prognostic marker because of the expression of this protein in various cancer cells [72] including oral squamous cell carcinoma [73] and prostate cancer [74,75,76]. It was found that Annexin A2 is highly expressed in breast tumor tissues [77] and that FOXD1-dependent RalA-ANXA2-Src complex promotes circulating tumor cell formation in BC [78]. Moreover, Annexin A5 (ANXA5) was found to promote prostate cancer stem cells, pancreatic adenocarcinoma, sarcoma and tumorigenesis and progression of BC [79]. This protein has been proposed to be a predictive biomarker for tumor development, metastasis and invasion and be of diagnostic, prognostic and therapeutic significance in cancer [79]. Moreover, in BC cells, ANXA5 up-regulation suppresses Raf-1 and MEK1/2 expressions, ERK1/2 phosphorylation and Ras activation in MCF-7 [80]. The protein ANXA6 has been implicated in various cellular functions including cell growth, differentiation and motility, which underlie tumor progression [81]. Consequently, reduced expression of ANXA6 is associated with decreased cell motility and rapid growth of xenograft TNBC tumors in vivo [81]. Our work demonstrates the high level of acetylation of several annexins in cancerous BC derived EVs suggesting that this post-translational modification may cause a change in protein structure and function, and consequently alter its function.

Aberrant acetylated histones identified mainly in cancerous cell line derived sEVs include H4 clustered histone 9, H3 clustered histone 1, H3.4 histone, H2B clustered histone 18, H2B clustered histone 20 and H1.5 linker histone. Epigenetic regulation via histone acetylation plays an important role in chromatin remodeling and in the regulation of gene transcription, which can be involved in tumorigenesis [53,82]. Several studies have suggested modifications to histone acetylation and proposed them as potential diagnostic or prognostic biomarkers in cancer [53]. Analysis of global histone modification in BC patients’ analysis revealed that the loss of acetylated H4 at K16 may serve as an early indication of cancer, and low levels of H3 acetylated at K9 and K14, and H4 at K12 are prognostic of poor outcomes [83]. In our work we identified the acetylation of H2B clustered histone 18 in both MCF7 and MDA-MB-231 derived EVs. It has been shown that the expression profile of MAP3K4-deficient trophoblast stem cells includes an H2B acetylation-regulated gene signature that closely overlaps with that of human BC cells [84].

In our previously published works [8,16], we investigated potential enzyme biomarkers for BC early detection, exploring the proteomic content of sEVs from cancerous cell lines. The discovery of potential biomarkers based on enzymatic assays with reliable clinical significance would most likely require a panel of multiple enzymes. For this reason, future studies to find more enzyme candidates should be performed using proteomics approaches on different cell lines as well as isolated blood from healthy and BC patients. Our results, based on proteomics analysis of sEVs from cancerous cell lines, found that three enzymes, ALDOA, PGK1 and ENO, may be potential biomarkers for BC diagnosis. These enzymes were already investigated to significantly increase activity in BC cells and tissue [59,60] supporting future exploration of these enzymes for BC prognosis.

## 5. Conclusions

Here, we performed a protein acetylation analysis of breast cancer-derived extracellular vesicles from MCF10A, MCF7 and MDA-MB-231 cell lines. We found that 60 distinct acetylated proteins were identified collectively among cell lines, covering different metabolic functions and especially within the glycolytic pathway. Among these acetylated proteins, we validated five glycolytic enzymes, ALDOA, GAPDH, PGK1, ENO and PKM. The results revealed that the specific activity of GAPDH and PKM in cancerous cell lines was at the limit of detection and therefore cannot be taken in consideration as a viable biomarker. In contrast, acetylated enzymes ALDOA, PGK1 and ENO showed a significantly higher specific enzymatic activity in MDA-MB-231 in comparison to MCF10A-derived EVs. Our results, based on protein acetylation analysis of BC-derived sEVs as well as previously reported studies on BC cells or tissues, strongly suggest that these enzymes may be good candidates as potential prognostic biomarkers for BC.

## Figures and Tables

**Table 1:** Summary of the acetylation sites for enzymes related to the glycolysis pathway, TCA cycle, and acetyl-CoA metabolism from MCF10A, MCF7 and MDA-MB-231 sEVs. These acetylated enzymes were identified at least in two replicate samples for each cell line.

Enzyme	Gene	Acetylation Sites	MCF10A sEV	MCF7 sEV	MDA-MB-231 sEV
			Position of Acetylation		
Aldolase	ALDOA	3	K-147	K-42, K-147, K-230	K-147, K-230
Glyceraldehyde-3-P-dehydrogenase	GAPDH	3		K-219	K-61, K-194, K-219
Phosphoglycerate kinase 1	PGK1	1			K-131
phosphoglycerate mutase 1	PGAM1	1			K-100
Enolase 1	ENO1	4	K-343	K-343	K-60, K-193, K-203, K-343
Enolase 2	ENO2	1			K-343
Enolase 3	ENO3	2			K-60, K394
pyruvate kinase M1/2	PKM	1		K-433	K-433

## Data Availability

The raw MS data presented in this study is openly available at the PRIDE repository. This data can be found here: https://www.ebi.ac.uk/pride/archive/projects/PXD040413.

## Supplementary Materials

The following supporting information can be downloaded at: https://www.mdpi.com/article/10.3390/biomedicines11041076/s1, Figure S1: Exo-Check exosome antibody array analysis for expression of well-known five external (CD63, EpCAM, ANXA5, CD81 and ICAM) and three internal (TSG101, ALIX and FLOT1) EV protein exosomal (or sEV) markers isolated from MCF10A, MCF7 and MDA-MB-231 cells. GM130 cis-Golgi marker is used to monitor any cellular contamination in sEV isolations, a labeled positive control for HRP detection, and a blank spot as a background control; Figure S2: The position of annexin acetylation sites from MCF10A, MCF7 and MDA-MB-231 marked with blue, red and yellow, respectively; Figure S3: The position of acetylated sites of enzymes in the glycolysis pathway from MCF10A, MCF7 and MDA-MB-231, respectively; Table S1: A total number of identified acetylated peptides for each sEV sample; Table S2: The list of identified acetylated proteins from three cell lines derived sEV; Table S3: The list of acetylated sites identified from three cell lines derived sEV; Table S4: GO functional annotation labels for cellular compartment and KEGG pathway analysis for sEVs from MCF10A, MCF7 and MDA-MB-231; Table S5: The specific activities of ALDOA, GAPDH, PGK1, ENO and PKM in the different cell free extracts and their derived sEV fractions. The numbers in parentheses indicate the number of biological replicates.
