# Supplementary material for: Lysine Acetylome of Breast Cancer-Derived Small Extracellular Vesicles Reveals Specific Acetylation Patterns for Metabolic Enzymes
Source: Biomedicines. 2023 Apr 2;11(4):1076. doi: 10.3390/biomedicines11041076 (PMC10135746; doi:10.3390/biomedicines11041076)
Supplement: Supplementary file 1 [file biomedicines-11-01076-s001.zip › biomedicines-2291775-supplementary-Figures S1-S3.pdf]

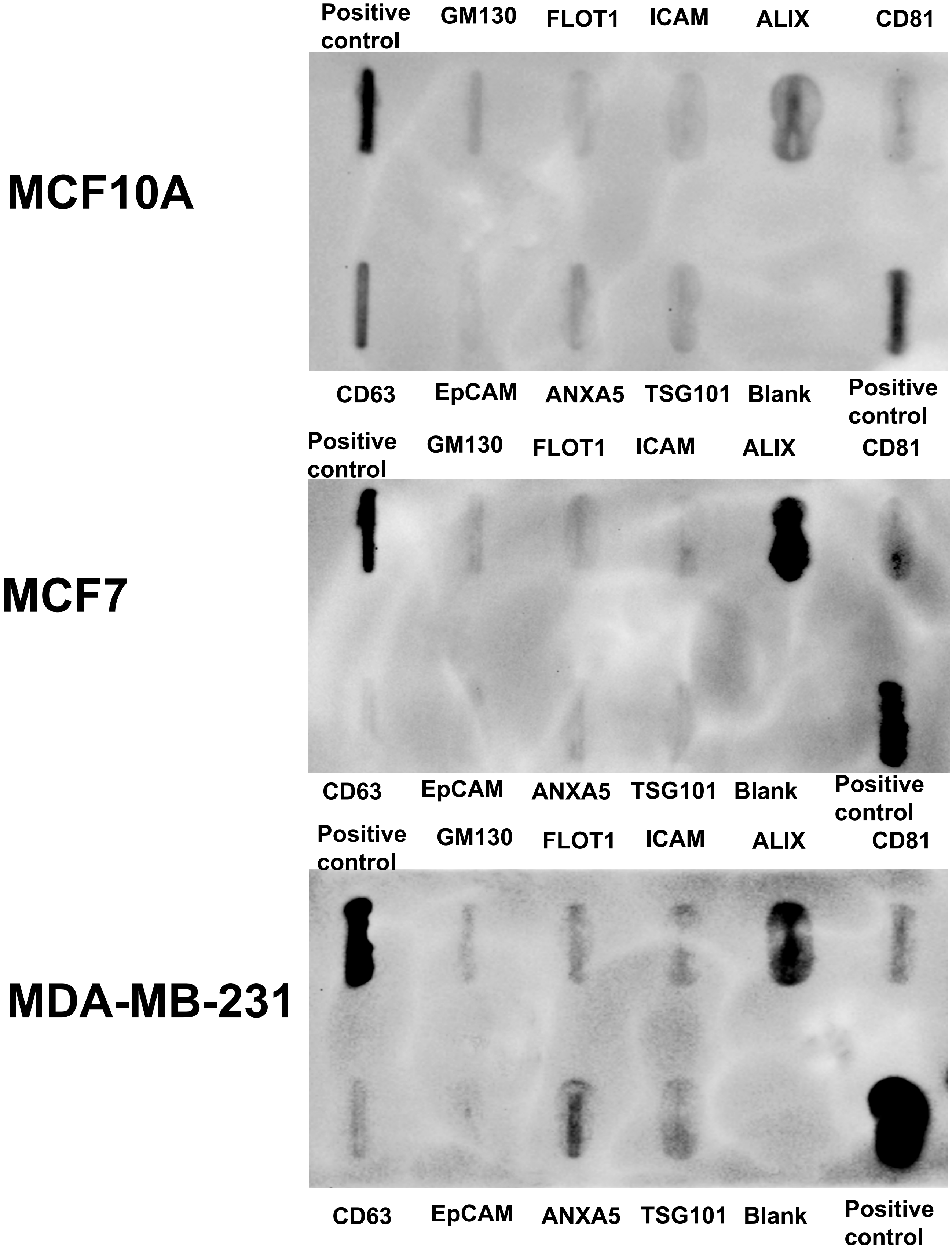

Figure S1: Exo-Check exosome antibody array analysis for expression of five external (CD63, EpCAM, ANXA5, CD81, and ICAM) and three internal (TSG101, ALIX, and FLOT1) EV protein exosomal (or sEV) markers isolated from MCF10A, MCF7 and MDA-MB-231 cells. GM130 cis-Golgi marker is used to monitor any cellular contamination in sEV isolations, a labeled positive control for HRP detection, and a blank spot as a background control.

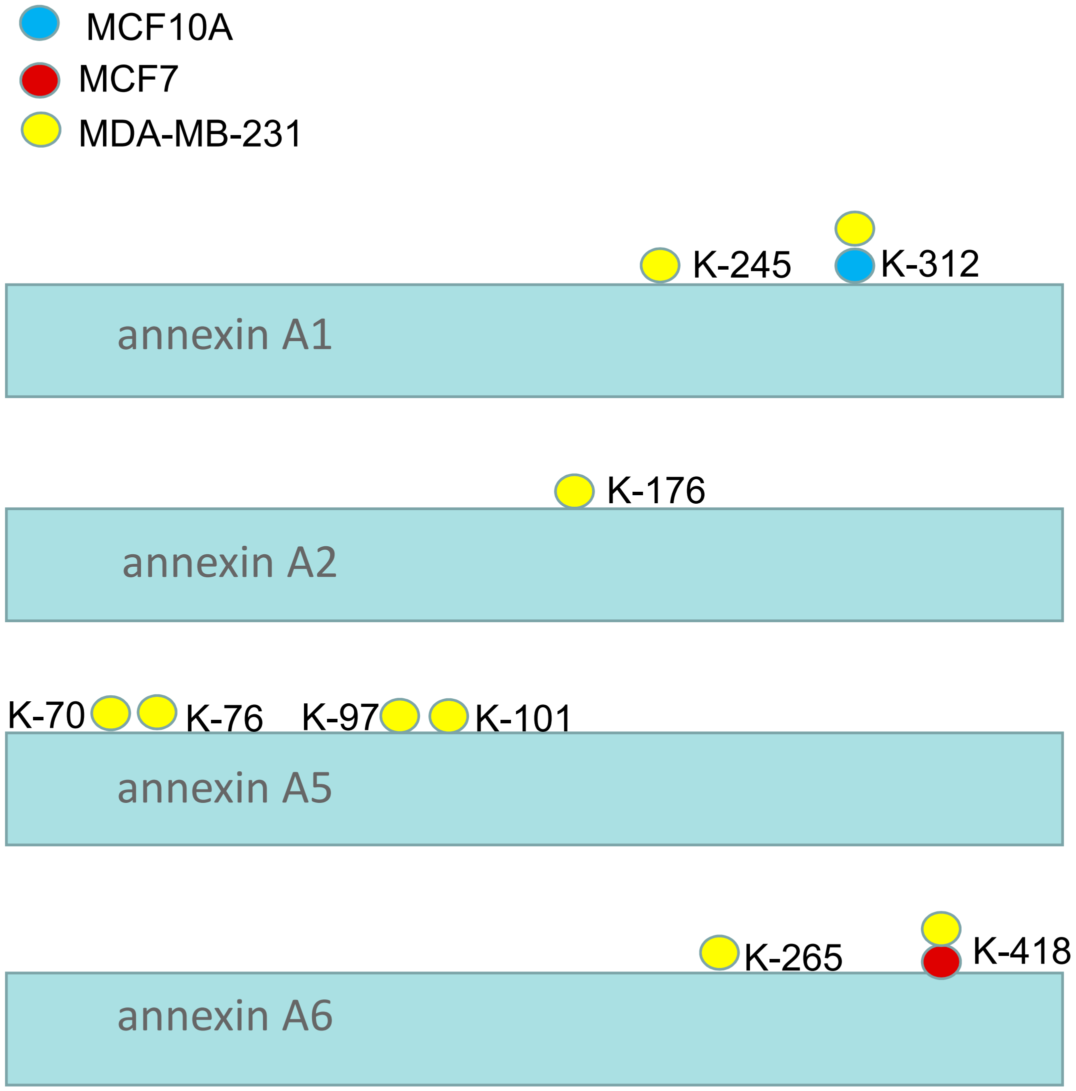

Figure S2: The position of annexin acetylation sites from MCF10A, MCF7 and MDA-MB-231 marked with blue, red and yellow, respectively.

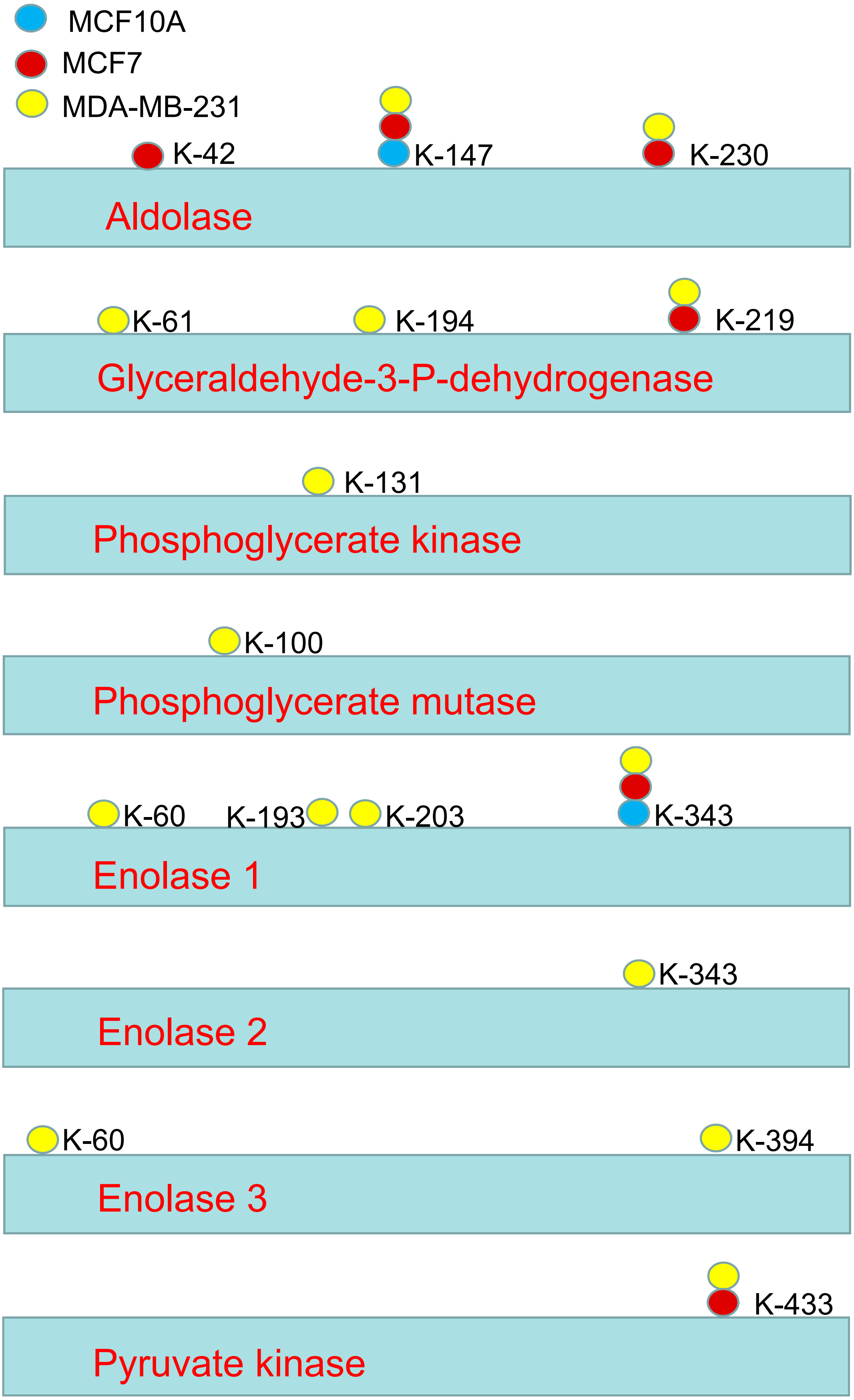

Figure S3: The position of acetylated sites of enzymes in the glycolysis pathway from MCF10A, MCF7 and MDA-MB-231, respectively.
